# Identification and Expression Analysis of Four Small Heat Shock Protein Genes in Cigarette Beetle, *Lasioderma serricorne* (Fabricius)

**DOI:** 10.3390/insects10050139

**Published:** 2019-05-15

**Authors:** Wen-Jia Yang, Kang-Kang Xu, Yu Cao, Yong-Lu Meng, Yan Liu, Can Li

**Affiliations:** Guizhou Provincial Key Laboratory for Rare Animal and Economic Insect of the Mountainous Region, College of Biology and Environmental Engineering, Guiyang University, Guiyang 550005, China; yangwenjia10@126.com (W.-J.Y.); kkxu1988@163.com (K.-K.X.); yucaosuccess@126.com (Y.C.); mengyonglu@126.com (Y.-L.M.); gyly68@sina.com (Y.L.)

**Keywords:** *Lasioderma serricorne*, small heat shock protein, development, stress responses, expression pattern

## Abstract

Small heat shock proteins (sHsps) are molecular chaperones that play crucial roles in the stress adaption of insects. In this study, we identified and characterized four *sHsp* genes (*LsHsp19.4*, *20.2*, *20.3*, and *22.2*) from the cigarette beetle, *Lasioderma serricorne* (Fabricius). The four cDNAs encoded proteins of 169, 180, 181, and 194 amino acids with molecular weights of 19.4, 20.2, 20.3, and 22.2 kDa, respectively. The four *LsHsp* sequences possessed a typical sHsp domain structure. Quantitative real-time PCR analyses revealed that *LsHsp19.4* and *20.3* transcripts were most abundant in pupae, whereas the transcript levels of *LsHsp20.2* and *22.2* were highest in adults. Transcripts of three *LsHsp* genes were highly expressed in the larval fat body, whereas *LsHsp20.2* displayed an extremely high expression level in the gut. Expression of the four *LsHsp* genes was dramatically upregulated in larvae exposed to 20-hydroxyecdysone. The majority of the *LsHsp* genes were significantly upregulated in response to heat and cold treatments, while *LsHsp19.4* was insensitive to cold stress. The four genes were upregulated when challenged by immune triggers (peptidoglycan isolated from *Staphylococcus aureus* and from *Escherichia coli* 0111:B4). Exposure to CO_2_ increased *LsHsp20.2* and *20.3* transcript levels, but the *LsHsp19.4* transcript level declined. The results suggest that different *LsHsp* genes play important and distinct regulatory roles in *L. serricorne* development and in response to diverse stresses.

## 1. Introduction

Heat shock proteins (Hsps), a group of molecular chaperones, play important roles in promoting correct refolding and blocking aggregation of denatured proteins [1]. Hsps represent a large gene superfamily and are universally present in the majority of living organisms ranging from bacteria to mammals. Hsps are stress-related proteins that are highly expressed in response to external stresses, including exposure to extreme temperatures [2,3], ultraviolet radiation [4], heavy metals [5], parasitic infection [6], and chemicals [7], as well as in response to starvation [8] and oxidation [9]. In addition, Hsps exhibit a variety of biological functions in early embryogenesis, diapause, and morphogenesis [10]. On the basis of their molecular mass and sequence similarities, Hsps have been classified into six families: Hsp100, Hsp90, Hsp70, Hsp60, Hsp40, and small Hsps [11,12]. Among these families, small Hsps are probably the most diverse protein family and show the greatest variation in sequence, size, and function [13].

Small heat shock proteins (sHsps) range in molecular mass from approximately 12 to 43 kDa [14]. Typically, sHsps are relatively conserved in amino acid structure and composition. The sequence generally contains an α-crystallin domain of 80–100 amino acids located near the C-terminal region, a disorganized N-terminus, and a variable C-terminus [15,16]. Most sHsps display chaperone-like activities, preventing aggregation and facilitating the correct refolding of denatured proteins [17,18]. Apart from their fundamental functions in stressful conditions, sHsps also participate in other physiological processes, including cell growth, differentiation, apoptosis [19], membrane fluidity [20], lifespan [21], and diapause [22]. At present, several sHsp cDNA sequences have been identified in numerous insect species. For example, 10 *sHsp* genes are known in *Apis mellifera*, seven in *Anopheles gambiae*, 11 in *Drosophila melanogaster*, 10 in *Tribolium castaneum*, and 16 in *Bombyx mori* [23], 14 in *Plutella xylostella* [24], 15 in *Choristoneura fumiferana* [25], and five in *Bemisia tabaci* [26]. Multiple *sHsp* genes have diverse and variable functions during insect growth and development. Previous studies have demonstrated that sHsps play essential roles in insect development and in defense against a variety of stresses. For instance, sHsps contribute to thermal tolerance in *Laodelphax striatellus* [27]. The expression of three *sHsp* genes in flesh fly (*Sarcophaga crassipalpis*) is upregulated during overwintering pupal diapause [28]. In *D. melanogaster*, overexpression of *hsp22* increases resistance to oxidative stress and aging [29]. RNA-interference experiments in *T. castaneum* indicate that knockdown of *Tchsp18.3* affects pupal-adult metamorphosis and reduces adult reproduction [30]. In *Apis cerana*, silencing of *sHsp22.6* significantly decreases temperature tolerance, and the recombinant sHsp22.6 protein exhibits remarkable temperature tolerance, antioxidation, and molecular chaperone activities [31].

The cigarette beetle, *Lasioderma serricorne* (Fabricius) (Coleoptera: Anobiidae), is a destructive and economically important storage pest worldwide [32]. Outbreaks of this species constitute a severe threat to many stored products, including cereals, tobacco, dry foods, and traditional Chinese medicinal materials [33,34]. After egg hatching, *L. serricorne* larvae bore tunnels into the stored materials and spend their larval stage inside host products [35,36]. Control of *L. serricorne* has depended heavily on the application of chemical insecticides. Methyl bromide, phosphine, pyrethrin, and organophosphorus are the primary compounds used for cigarette beetle management [37,38]; however, the excessive use of chemicals has resulted in resistance development and environmental contamination [39,40]. Extreme temperature treatments and low-oxygen controlled atmospheres have previously shown promise as ecologically friendly alternatives to conventional control methods [41,42]; however, *L. serricorne* has developed substantial tolerances to extreme temperatures and oxygen-deficit conditions [43]. The high adaptability of *L. serricorne* in response to various stresses renders it difficult to control. However, insect sHsps could be used to modify the biological responses elicited by external abiotic stresses. To date, no study has investigated the effects of stresses on sHsps in *L. serricorne* at the molecular level.

In the present study, we identified and cloned the full-length open reading frame (ORF) sequences of four *sHsp* genes in *L. serricorne*. We analyzed the expression patterns of the *sHsps* in different developmental stages and tissues, and in response to 20-hydroxyecdysone (20E) treatment. In addition, we evaluated the responses of the four *sHsps* to diverse stresses, comprising thermal stress, immune challenges, and CO_2_ stress.

## 2. Materials and Methods

### 2.1. Insect and Sample Preparation

The laboratory stock colony of *L. serricorne*, originally collected in 2014 from a tobacco warehouse in Guizhou Province, China, was reared on Chinese medicinal material (*Angelica sinensis*) and maintained at 28 ± 1 °C and 40% ± 5% relative humidity under a scotoperiod of 24 h.

Samples at different developmental stages, including early larvae (EL, <24 h post-hatching), late larvae (LL, older than fourth instar larvae and before prepupae), pupae (PU, >48 h post-pupation), early adults (EA, <24 h post-eclosion), and late adults (LA, one week old) were collected separately and stored at −80 °C. In the tissue-specific experiment, the fifth instar larvae were used for tissue isolation. The integument, fat body, gut, and carcass of *L. serricorne* were dissected under a stereomicroscope (Olympus SZX12, Tokyo, Japan). Each tissue type was placed in a 1.5-mL centrifuge tube containing RNA storage reagent (Tiangen, Beijing, China). Pools of 30 individuals of larvae were used to prepare the integument, gut, and carcass, and 50 individuals were pooled to collect the fat body tissue. All tissue samples were immediately frozen in liquid nitrogen and stored at −80 °C. Each sample was replicated three times.

### 2.2. 20E and Temperature Treatment

For the 20E (Sigma-Aldrich, St. Louis, MO, USA) treatment, a 10 μg/μL stock solution of 20E dissolved in 95% ethanol was diluted to 1 μg/μL with distilled water and used as the working solution. For the treatment group, fourth instar larvae were injected at a dose of 120 ng/larva using a Nanoliter 2010 injector (World Precision Instruments, Sarasota, FL, USA). The control group was injected with an equivalent volume of distilled water containing 0.1% ethanol. At 4, 8, and 12 h after injection, the whole bodies of surviving insects were frozen in liquid nitrogen and stored at −80 °C. For the temperature treatment, the early female and male adults were placed in small glass tubes and exposed to a range of temperatures (5, 40, 42, 44, and 46 °C) for 2 h in a temperature-controlled chamber (DC-3100, Ningbo, China). After exposure, adults were allowed to recover at 28 °C for 2 h, and survivors were frozen in liquid nitrogen and stored at −80 °C. A set of adults maintained at 28 °C was used as a control. At least 30 insects were randomly collected per replication, and three independent biological replications were performed.

### 2.3. Immune Challenge and Controlled Atmosphere Treatment

For the immune challenge, peptidoglycan from *Staphylococcus aureus* (PGN-SA; InvivoGen, San Diego, CA, USA) and peptidoglycan from *Escherichia coli* 0111:B4 (PGN-EB; InvivoGen) were diluted with sterile endotoxin-free water to a final concentration of 1.0 μg/μL. The fourth instar larvae were injected with 200 nL of PGN-SA solution, PGN-EB solution, or sterile endotoxin-free water. The control group was handled in the same manner but without injection. Thirty individuals were randomly selected from each group after 3, 6, and 9 h post-injection. For the controlled atmosphere (CA) treatment, adults were exposed to air containing 70% CO_2_ for 6 h. The treated insects were then transferred to natural atmospheric conditions for 6 h, and the mortality rate was recorded as 35.81%. The control group was maintained under natural atmospheric conditions. Approximately 40 surviving individuals were randomly selected from each group. Each of the above-mentioned treatments included three biological replications. All samples were frozen in liquid nitrogen and stored at −80 °C for RNA extraction.

### 2.4. RNA Extraction and cDNA Synthesis

Total RNA was extracted from each sample using the MiniBEST Universal RNA Extraction Kit (TaKaRa, Dalian, China) and treated in a gDNA Eraser spin column for genomic DNA removal. The RNA quantity was measured using a NanoDrop 2000C spectrophotometer (Thermo Fisher Scientific, Waltham, MA, USA) with absorbance levels of 260 nm and 280 nm. The RNA integrity was verified by 1% agarose gel electrophoresis. The cDNA for cloning and quantitative real-time PCR (qPCR) was synthesized using 1 μg RNA from each sample using the PrimeScript^®^ RT Reagent Kit (TaKaRa) with random hexamer and oligo (dT) primers.

### 2.5. Identification and Sequencing of LsHsp cDNAs

Based on the *L. serricorne* transcriptome database (unpublished data), four unigene cDNAs encoding *sHsps* were obtained. The full-length ORFs of the four genes were confirmed by PCR with the corresponding pair of specific primers (Table 1). The PCR reaction was as follows: 95 °C for 3 min; followed by 35 cycles at 95 °C for 30 s, 55–65 °C (depending on the gene-specific primers) for 30 s, and 72 °C for 1 min, and then a final extension for 10 min at 72 °C. The PCR products were separated by 1% agarose gel electrophoresis and sub-cloned into the pGEM-T Easy vector (Promega, Madison, WI, USA) for sequencing.

### 2.6. Bioinformatics and Phylogenetic Analyses

The ORFs were predicted with ORF finder (http://www.ncbi.nlm.nih.gov/gorf/gorf.html). DNAMAN 6.0 (LynnonBiosoft, Vaudreuil, Quebec, Canada) was used to generate a multiple-sequence alignment. Sequence similarities and the presence of conserved domains were determined using the Basic Local Alignment Search Tool algorithm-based programs available on the National Center for Biotechnology Information website (http://www.blast.ncbi.nlm.nih.gov/Blast). The molecular weights and isoelectric points were predicted with ExPASy (http://us.expasy.org/tools/protparam.html). Secondary structure predictions were performed with PHD software accessed through the NPS@Web server (http://npsa-pbil.ibcp.fr) [44]. A phylogenetic analysis was performed by MEGA 6.06 [45] using the neighbor-joining method with 1000 bootstrap replicates and pairwise deletion.

### 2.7. Quantitative Real-Time PCR

The qPCR analysis was performed using the CFX-96 real-time PCR System (Bio-Rad, Hercules, CA, USA) in a total reaction volume of 20 μL, including 10 μL of GoTaq^®^ qPCR Master Mix (Promega), 1 μL of cDNA template, 1 μL of each of the gene-specific primers, and 7 μL of nuclease-free water. All primers used for qPCR were designed using Primer 3 (version 0.4.0) software (http://fokker.wi.mit.edu/primer3/) (Table 1). The reaction procedure was performed as follows: 95 °C for 2 min, followed by 40 cycles of 95 °C for 30 s and 60 °C for 30 s. A melting curve analysis from 60 °C to 95 °C was applied to all reactions to ensure the specificity and consistency of the generated products. All experiments were performed in triplicate, with two technical replicates each. The 18S ribosomal RNA (*18S*) gene was used as an internal reference gene. The relative expression level of the *sHsp* genes was calculated using the 2^−ΔΔCt^ method [46].

### 2.8. Data Analyses

For all statistical analyses, IBM SPSS Statistics version 20.0 software (IBM Corporation, Armonk, NY, USA) was used. The expression data were presented as the mean ± standard error. Significant differences among treatments were analyzed by one-way analysis of variance (ANOVA) followed by a least significant difference test for multiple comparisons or a two-tailed, unpaired *t*-test for comparisons of two means.

## 3. Results

### 3.1. Identification and Characterization of Four LsHsp Genes

Four *LsHsp* genes, namely *LsHsp19.4*, *20.2*, *20.3*, and *22.2* (GenBank accession numbers: MK395540, MK395541, MK395542, and MK395543) were cloned from *L. serricorne*. Sequencing analysis revealed that the cDNAs included ORFs of 510, 543, 546, and 585 bp, respectively, encoding proteins of 169, 180, 181, and 194 amino acids, respectively. The predicted molecular weights of the LsHsps ranged from 19.4 to 22.2 kDa, with theoretical isoelectric points of 5.94 to 6.60 (Table 2).

**Figure 1:**
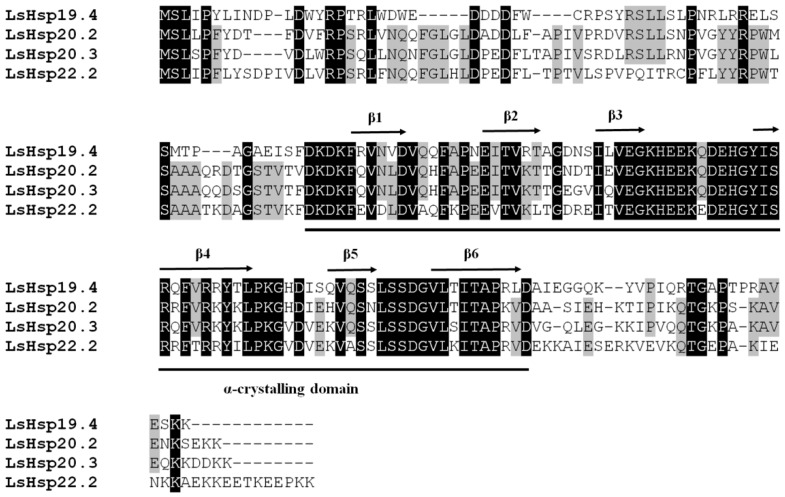
Alignment of the deduced amino acid sequences of four *LsHsp* genes in *L. serricorne*. Amino acids with >50% identities are shaded in gray. Sequences above the black lines are regions of *α*-crystallin domain. Six *β*-strands with the *α*-crystallin domain are indicated with black arrows.

The amino acid residue identities were 20%–63% among the four LsHsps. Multiple alignments of the deduced amino acid sequences revealed that the four sHsp proteins contained a typical *α*-crystallin domain, which consisted of approximately 100 amino acids and six *β*-strands (Figure 1).

**Figure 2:**
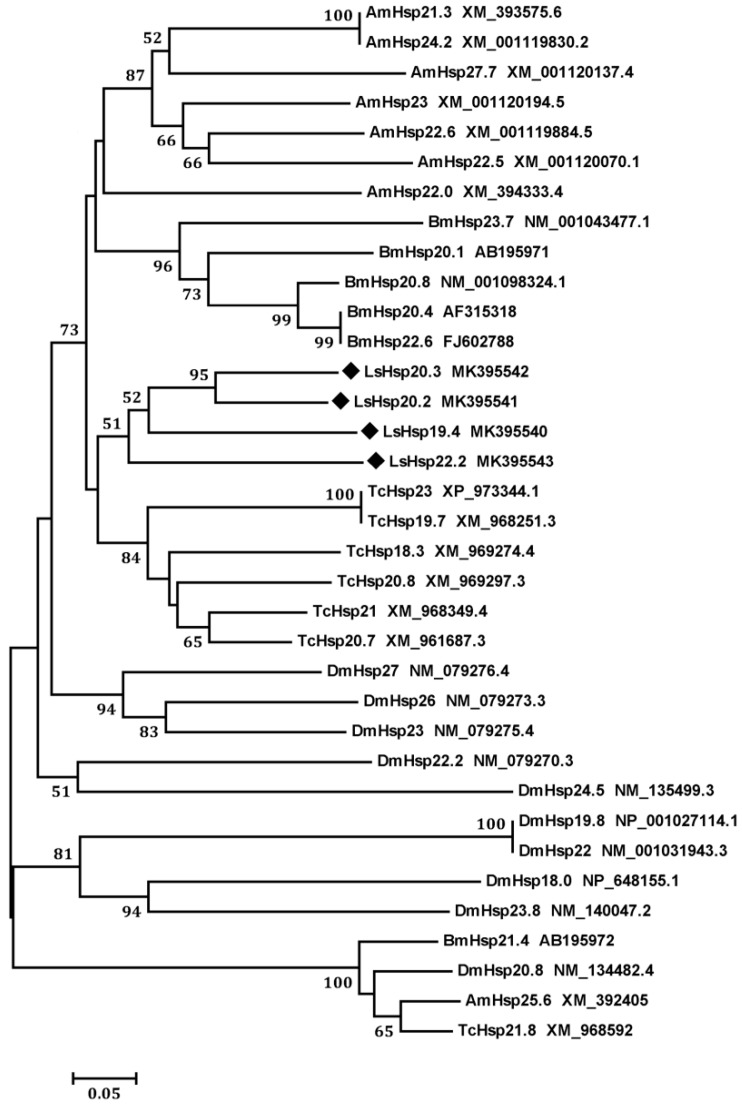
Phylogenetic analysis of small heat shock proteins (sHsps) in *L. serricorne* and other insects. The tree was generated with MEGA6.06 using the neighbor-joining method. Nodes with >50% bootstrap values (1000 replications) are indicated on branches. The following insect *sHsp* sequences were used: *Apis mellifera* (Am), *Bombyx mori* (Bm), *Drosophila melanogaster* (Dm), *Lasioderma serricorne* (Ls), *Tribolium castaneum* (Tc). The GenBank accession number for each sequence is specified in the terminal label. The *sHsp* sequences determined in this study are labeled with a black diamond.

To analyze the relatedness of the four LsHsps with sHsps from other insect species, we constructed a phylogenetic tree using the neighbor-joining method. The four LsHsps (LsHsp19.4, 20.2, 20.3, and 22.2) showed high sequence similarity to each other and were grouped into one cluster (Figure 2).

### 3.2. Expression Levels of Four LsHsp Genes at Different Developmental Stages

**Figure 3:**
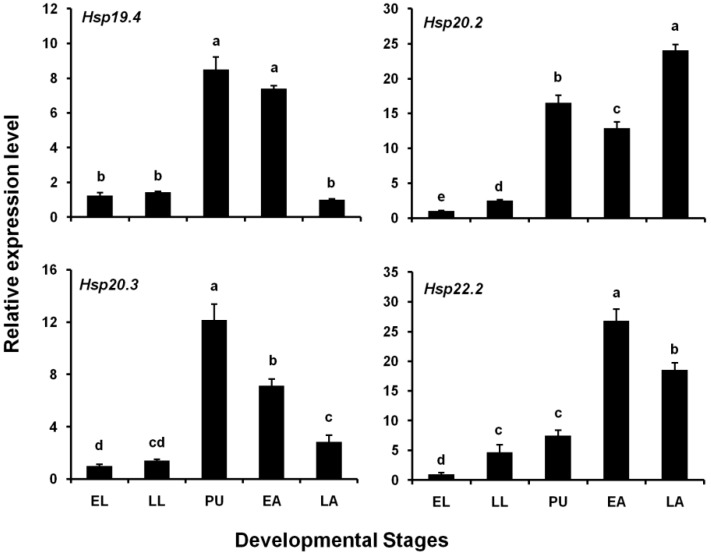
Relative expression levels of four *LsHsp* genes at different developmental stages of *L. serricorne*. EL, early larvae; LL, late larvae; PU, pupae; EA, early adults; LA, late adults. Different letters above bars indicate a significant difference among developmental stages based on one-way ANOVA followed by a least significant difference test (*p* < 0.05).

The expression levels of the four *LsHsp* genes at different developmental stages (early larvae, late larvae, pupae, early adults, and late adults) were estimated by qPCR. Generally, the four genes were consistently expressed at all tested stages, but exhibited different developmental expression patterns (Figure 3). The highest expression levels of *LsHsp19.4* and *20.3* were observed in pupae and were respectively 8.49- and 12.2-fold greater than those at other stages. The maximum expression level of *LsHsp20.2* was recorded in late adults and was 24.1-fold greater than those at other stages. *LsHsp22.2* transcripts were most abundant in early adults and were 26.9-fold higher than those at other stages.

### 3.3. Expression Levels of Four LsHsp Genes in Different Tissues

**Figure 4:**
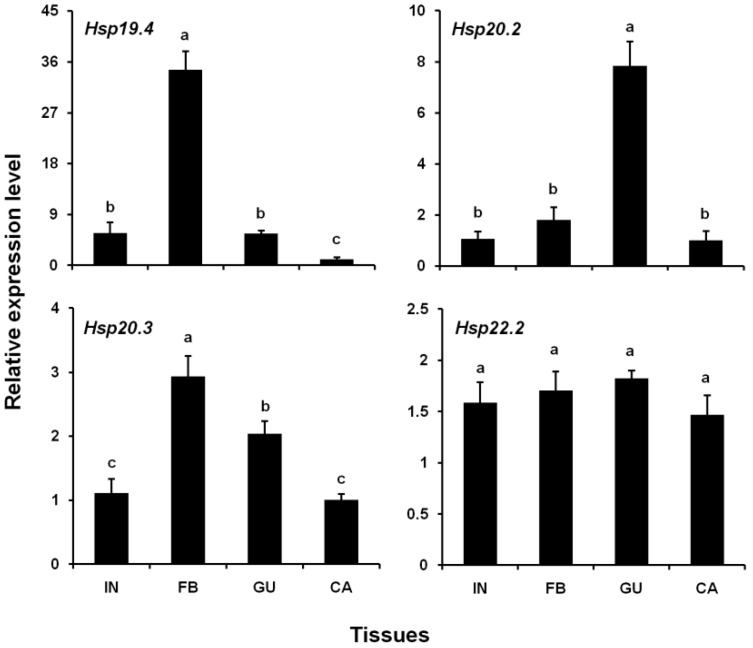
Relative expression levels of four *LsHsp* genes in different tissues of *L. serricorne*. IN, integument; FB, fat body; GU, gut; CA, carcass. Different letters above bars indicate a significant difference among tissues based on one-way ANOVA followed by a least significant difference test (*p* < 0.05).

All four *LsHsp* genes were expressed in the four tissue types analyzed. Gene expression varied significantly with tissue type except for *LsHsp22.2*. Interestingly, the highest expression levels of *LsHsp19.4* and *LsHsp20.3* were detected in the fat body, while the lowest expression levels were recorded for the carcass (Figure 4). Additionally, the maximum expression level of *LsHsp20.2* was observed in the gut, which was 6.17-fold greater than that detected in the other tissues.

### 3.4. Effects of 20E Treatment on the Expression Levels of Four LsHsp Genes

**Figure 5:**
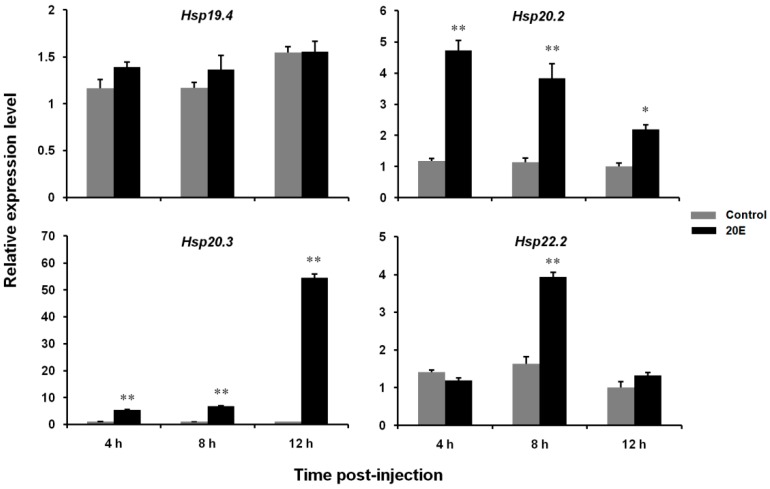
Relative expression levels of four *LsHsp* genes in *L. serricorne* in response to 20E induction. Larvae were collected for qPCR analysis at 4, 8, and 12 h after injection with 20E. The corresponding amount of distilled water containing 0.1% ethanol was used as a control. Significant differences between treatment and control were determined using a *t* test and indicated by * (*p* < 0.05) or ** (*p* < 0.01).

To determine whether 20E induces *LsHsp* expression in vivo, the mRNA levels of the four *LsHsp* genes in the fourth instar larvae were quantified in response to 20E treatment. The transcript levels of *LsHsp20.2* and *20.3* increased significantly after injection of 20E at all three time points, but the response of *LsHsp20.3* was much more intense than that of *LsHsp20.2*. Compared with the control specimens, the expression level of *LsHsp22.2* dramatically increased in larvae injected with 20E at 8 and 12 h. However, 20E-induced expression was not observed for *LsHsp19.4* (Figure 5).

### 3.5. Expression Levels of Four LsHsp Genes in Response to Thermal Stress

**Figure 6:**
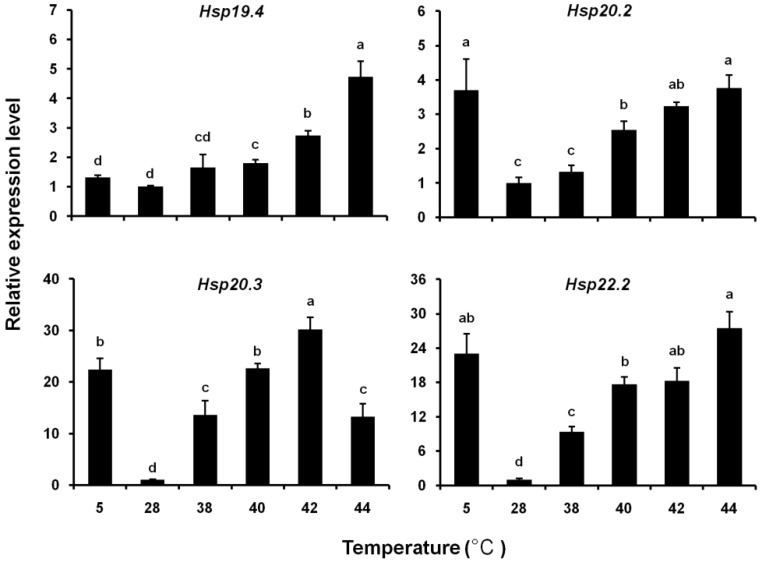
Relative expression levels of four *LsHsp* genes in *L. serricorne* in response to thermal stress. Different letters above bars indicate a significant difference based on one-way ANOVA followed by a least significant difference test (*p* < 0.05).

The expression patterns of the four *LsHsp* genes in response to thermal stress differed substantially. During cold-shock treatment, the expression levels of three *LsHsp* genes were remarkably upregulated, whereas that of *LsHsp19.4* was not significantly altered. During heat-shock treatment, the expression levels of all four *LsHsp* genes were significantly increased, and the greatest induced expression of *LsHsp20.3* was observed in response to the 42 °C treatment (Figure 6). For *LsHsp20.3*, the response patterns initially increased and then decreased as the temperature increased. The other three *LsHsp* genes exhibited similar expression patterns that increased in a temperature-dependent manner.

### 3.6. Expression Levels of Four LsHsp Genes in Response to Immune Challenges

**Figure 7:**
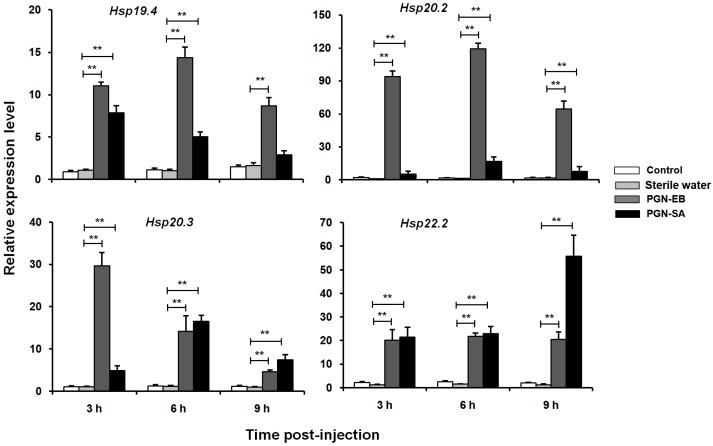
Relative expression levels of four *LsHsp* genes in *L. serricorne* after PGN-EB and PGN-SA challenges. Control: pricked with the micro-injection needle without injection; Sterile Water: injected with 200 nL sterile endotoxin-free water; PGN-SA: injected with 200 nL peptidoglycan from *Staphylococcus aureus* (PNA-SA) solution; PGN-EB: injected with 200 nL peptidoglycan from *Escherichia coli* 0111:B4 (PNA-EB) solution. Significant differences were determined using a *t* test and indicated by ** (*p* < 0.01).

To evaluate the responses of the *LsHsp* genes to immune challenges, the larvae were exposed to PGN-SA and PGN-EB at three time intervals (Figure 7). After PGN-EB exposure, expression levels of the four *LsHsp* genes were significantly increased by 1.34- to 119.1-fold. The maximum induced expression occurred in *LsHsp20.2* at 6 h after injection with PGN-EB. Injection of PGN-SA also significantly induced expression of *LsHsp* genes at 3, 6, and 9 h post-treatment. In addition, the induced expression levels of *LsHsp19.4* and *20.2* were greater in PGN-EB-treated groups than those in PGN-SA-treated groups.

### 3.7. Effects of CO_2_ Exposure on the Expression Levels of Four LsHsp Genes

**Figure 8:**
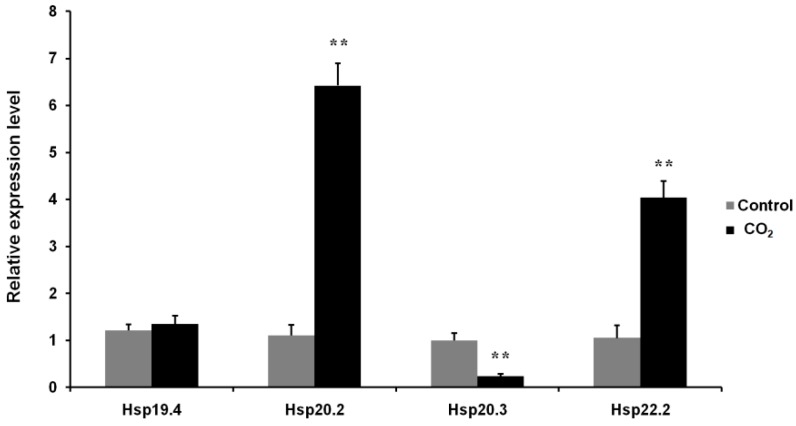
Relative expression levels of four *LsHsp* genes in *L. serricorne* in response to CO_2_ stress. Adults were collected for qPCR analysis at 6 h after exposure to air containing 70% CO_2_. The control group was maintained under natural atmospheric conditions. Significant differences between the treatment and control were determined using a *t* test and indicated by ** (*p* < 0.01).

To investigate the effects of CO_2_ exposure on *LsHsps* expression, adults were exposed to air containing 70% CO_2_ for 6 h. The expression levels of *LsHsp22.2* and *20.2* were significantly upregulated by 2.66- and 5.84-fold, respectively. However, the expression level of *LsHsp20.3* decreased by 4.2-fold after exposure to the CO_2_ treatment compared with that of the control (Figure 8). No difference in *LsHsp22.2* expression was observed in the CO_2_-treated group compared with that of the control.

## 4. Discussion

Insects possess different types of sHsps, which differ in structure and function among species [23,24,27,47]. The sHsps are known to play major roles in cell response to different stresses [48]. Analyses of these sHsps would provide valuable information for further functional studies in different insects. In the current study, we identified and cloned four *sHsp* genes (*LsHsp19.4*, *20.2*, *20.3*, and *22.2*) in *L. serricorne* based on our previous transcriptome data. Sequence analysis indicated that the four *sHsps* showed high similarity with other insect sHsps. The deduced amino acid sequences of four *LsHsps* contained a conserved *α*-crystalline domain consisting of common *β*-strands, which agrees with previous studies of sHsps in other insect species [47,49,50,51].

Insect sHsps play important roles in the regulation of development. In the current study, we observed that the expression of four *LsHsps* occurred in tested developmental stages, but these *LsHsps* showed distinct expression patterns. The mRNA levels of *LsHsp19.4* and *20.3* increased greatly when larvae molted into the pupal stage, suggesting that these two *sHsps* may have crucial functions in pupal formation. Similar expression patterns have been observed in other insect species, for example, *Hsp23*, *26*, and *27* in *D. melanogaster* [52], *Hsp19.5* in *P. xylostella* [53], *Hsp19.5*, *20.8*, and *21.7* in *Liriomyza sativae* [54], and *Hsp20.4* and *20.8* in *Spodoptera litura* [55]. In addition, *LsHsp 20.2* and *22.2* showed high expression levels during the adult stages. This finding is consistent with previous studies in which *sHsp* genes were upregulated in adults of *Chilo suppressalis* [49], *Cydia pomonella* [56], and *Harmonia axyridis* [57]. In *Sesamia inferens*, large quantities of *Hsp19.6* and *20.6* transcripts were observed in the egg [58]. Interestingly, *Hsp21.4* in *S. litura* and *Hsp21.6* in *Bactrocera dorsalis* exhibit constitutive expression patterns during all developmental stages [47,51]. Collectively, the diverse expression patterns of *sHsp* genes indicate that they might fulfill different roles during the developmental progression of insects.

The expression of insect sHsps shows distinct tissue specificity. In *B. mori*, *Hsp19.1* and *22.6* were highly expressed in the integument, head, and midgut, whereas *Hsp20.1*, *20.4*, and *27.4* exhibited high expression levels in the ovary and testis [23]. In *Oxya chinensis*, *Hsp19.1*, *20.4*, *20.7*, and *21.1* were selectively expressed in the ovary and testis, whereas *Hsp19.8* and *23.8* were mainly expressed in the muscle [50]. Previous studies showed that the Malpighian tubules are the primary site of *sHsp* expression, such as in *S. litura* [47], *C. suppressalis* [49], and *C. fumiferana* [25]. In the present study, *LsHsp19.4* and *20.3* were highly expressed in the fat body. Similar findings have been reported for other insect species, such as *B. mori* [59] and *B. dorsalis* [51]. The insect fat body is an important organ involved in diverse biological processes, including detoxification, immunity, energy metabolism, and nutrient storage. However, it remains unclear why *sHsps* were highly expressed in the fat body, and further studies are required to clarify their precise roles in *L. serricorne*. Similar to four *sHsp* genes (*Hsp19.5*, *20.1*, *21.6*, and *21.8*) from *P. xylostella* [24], *LsHsp20.2* showed a higher expression level in the gut than in other tissues analyzed. Interestingly, *LsHsp22.2* exhibited a constitutive expression pattern in the four tested tissues, suggesting that it may play fundamental roles in in vivo activities.

There is a functional correlation between hormones and heat-shock regulatory systems [60]. The expression of *sHsp* genes can be regulated by 20E in numerous insect species. Here, we found 20E upregulated mRNA levels of three *sHsp* genes in *L. serricorne* at different time points. This observation is in agreement with studies of *B. dorsalis* in which the expression levels of *BdHsp18.4*, *20.4*, and *20.6* were induced by 20E [51]. Similarly, significant upregulation of *SlHsp20.4* and *SlHsp20.8* by 20E was observed in the larval midgut and cell line of *S. litura* [47,55]. There is strong evidence for a connection between ecdysone and insect sHsps. For example, upregulation of *Hsp27* in *D. melanogaster* and *Ceratitis capitata* by 20E is mediated by a canonic ecdysone response element (EcRE) located in the promoter region [61,62]. Binding sites for the ecdysone-responsive transcription factor Broad-Complex (BR-C) were detected in the 5′-flanking regions of *Drosophila Hsp23* [63], *AccHsp23.0*, and *AccHsp24.2* in *A. cerana* [64]. Epidermis culture experiments showed that the expression of two *AccHsp* genes could be regulated by 20E [64]. Interestingly, expression of *SlHsp20.4* and *SlHsp20.8* was also induced by the juvenile hormone (JH), and the responses of two *sHsp* genes to JH induction were greater than that to 20E induction [55].

Studies of many *sHsp* genes in insects have demonstrated upregulated expression after thermal stress exposure. In the present study, three *LsHsp* genes were dramatically upregulated in response to cold treatment. Cold shock induces upregulation of *sHsp* genes, including *Hsp21.4*, *20.6*, and *19.6* in *S. inferens* [58], *Hsp20.4* and *20.8* in *S. litura* [47], and *Hsp19.8*, *21.5*, and *21.7b* in *C. suppressalis* [49]. Heat stress also induced the expression of four *sHsp* genes in *L. serricorne*, indicating their potential roles in heat resistance. Similar situations have been observed in several insects, including *B. mori* [23], *P. xylostella* [24], *B. dorsalis* [51], and *C. fumiferana* [25]. The sHsp bind to other cellular proteins under thermal stress and provide protection from denaturation. Different response patterns were observed for the four *sHsp* genes of *L. serricorne* in response to heat or cold treatment. Furthermore, *LsHsp19.4* was insensitive to cold stress, which is consistent with the responses of *Hsp20* and *21.4* in *S. litura* [47], and *Hsp17.7* and *21.6* in *B. dorsalis* [51]. Thus, different sHsps may have different mechanisms of action for adaptation to thermal stress.

It was reported that *sHsp* expression could be regulated by immune challenge in many insects. In *A. cerana*, for example, *AccsHsp22.6* expression was significantly increased by inoculation with the fungal pathogen *Ascosphaera apis* [31]. *AccsHsp27.6* expression was induced by exposure to *S. aureus* and *Micrococcus luteus* and suppressed by *Bacillus subtilis* and *Pseudomonas aeruginosa*. These results indicate that sHsps participate in the host immune response to different microbes, and additional analysis of recombinant Hsp27.6 protein provides antimicrobial evidence [9]. Treatment of *B. mori* larvae with cytoplasmic polyhedrosis virus (CPV) significantly increased the expression of *sHsp23.7*, suggesting its involvement in anti-BmCPV immunity [65]. Zhang et al. [66] reported that *sHsp20.8* expression in Chinese oak silkworm (*Antheraea perny*) was substantially upregulated after bacterial and viral infection. Recent studies of *T. castaneum* show that silencing of *Hsp18.7* amplifies the serine protease signaling pathway to regulate innate immunity of red flour beetles [67]. In the present study, the expression levels of four *LsHsp* genes were upregulated after treatment with Gram-positive peptidoglycan (PGN-SA) and Gram-negative peptidoglycan (PGN-EB), indicating that the *LsHsps* might play important roles in responses to different immune reactions. In addition, the expression of multiple insect *sHsp* genes is regulated by immune challenge. These effects are indirect and might be due to immune response elements [9,31].

Controlled atmosphere treatments using high CO_2_ (hypercapnia) and low oxygen (hypoxia) have been applied commercially to control the cigarette beetle [34,68]. However, several populations are reported to have developed substantial tolerance to evaluated CO_2_ [34]. Hypercapnia may cause many physiological consequences to insects, including the reduction of metabolic rate and initiation of anaerobic respiration [69]. Our previous studies showed that the specific activity of carboxylesterase in *L. serricorne* was increased following exposure to CO_2_-enriched atmosphere [34]. The expression of two glutathione *S*-transferase genes (*LsGSTt1* and *LsGSTs1*) could also be markedly induced by CO_2_ [70]. We concluded that detoxification enzymes may be critical for tolerance to CO_2_ stress. It has been demonstrated that sHsps are rapidly synthesized in cells after exposure to environmental stressors and develop protective functions. The sHsps are reported to be the best candidates for adaptation to various stresses. Studies of *A. cerana* show that *AccHsp27.6* expression is suppressed by CO_2_ treatment [9], which corresponds with the expression of *LsHsp20.3* observed in the present study. However, we found that transcription of *LsHsp20.2* and *20.3* responded differentially to CO_2_ treatment. These results suggest that different sHsps may play distinct roles in response to CO_2_ stress.

## 5. Conclusions

We identified and characterized four *sHsp* genes from *L. serricorne*. The putative sHsp proteins contained the typical structural and conserved domains of sHsps. The expression levels of these *sHsp* genes differed among various developmental stages and tissues. The expression levels of the four *LsHsp* genes were upregulated by 20E exposure. Induction of these genes in response to diverse stresses indicated that the genes play important roles in stress-adaptation mechanisms. Further studies are needed to clarify the precise roles and physiological mechanisms of the *sHsp* genes in *L. serricorne*.

## Figures and Tables

**Table 1:** Primers used in this study.

Application of Primers	Gene	Primer Sequence (5′ to 3′)
Full-length confirmation	*LsHsp19.4*	F: TGATAAACGACCCTCTGGAT R: ATGTACCCGTGTTCGTCTTG
	*LsHsp20.2*	F: CATTCTACGACACCTTTGAC R: AATGGAAGCTGCATCAACTT
	*LsHsp20.3*	F: TTGTCTCCATTCTACGACGT R: CCTTTTGGCAACTTATACTT
	*LsHsp22.2*	F: TTCCTATATTCCGACCCCAT R: TGATCTTCAACACACCATCA
qPCR analysis	*LsHsp19.4*	F: CTGGGAGGATGATGACGACT R: ACATCGACGTTCACACGAAA
	*LsHsp20.2*	F: ATTTCCAGGCGATTTGTGAG R: CTTTGCTTGGCTTTCCAGTC
	*LsHsp20.3*	F: AAACACGAGGAGAAGCAGGA R: CTGGTTTCCCAGTTTGCTGT
	*LsHsp22.2*	F: AAAGACGCTGGCTCAACTGT R: TCCTTTCGGCAGAATGTACC
	*18S*	F: GTTGATCACGTCGCAAGCTA R: AGGTTTCCCTCTGGCTTGTT

**Table 2:** Characteristics of *sHsp* mRNAs in *Lasioderma serricorne*.

Gene	Sequence Length (bp)	Protein Length (aa)	Molecular Weight (kDa)	Isoelectric Point	GenBank Accession Number
*LsHsp19.4*	510	169	19.4	5.94	MK395540
*LsHsp20.2*	543	180	20.2	6.60	MK395541
*LsHsp20.3*	546	181	20.3	6.32	MK395542
*LsHsp22.2*	585	194	22.2	6.04	MK395543
